# StrainIQ: A Novel *n*-Gram-Based Method for Taxonomic Profiling of Human Microbiota at the Strain Level

**DOI:** 10.3390/genes14081647

**Published:** 2023-08-18

**Authors:** Sanjit Pandey, Nagavardhini Avuthu, Chittibabu Guda

**Affiliations:** 1 Department of Genetics, Cell Biology and Anatomy, University of Nebraska Medical Center, Omaha, NE 68198, USA; 2 Center for Biomedical Informatics Research and Innovation, University of Nebraska Medical Center, Omaha, NE 68198, USA

**Keywords:** *n*-grams, StrainIQ, metagenomics, microbiota, DSEM, strain-level, site-specific

## Abstract

The emergence of next-generation sequencing (NGS) technology has greatly influenced microbiome research and led to the development of novel bioinformatics tools to deeply analyze metagenomics datasets. Identifying strain-level variations in microbial communities is important to understanding the onset and progression of diseases, host–pathogen interrelationships, and drug resistance, in addition to designing new therapeutic regimens. In this study, we developed a novel tool called StrainIQ (strain identification and quantification) based on a new *n*-gram-based (series of *n* number of adjacent nucleotides in the DNA sequence) algorithm for predicting and quantifying strain-level taxa from whole-genome metagenomic sequencing data. We thoroughly evaluated our method using simulated and mock metagenomic datasets and compared its performance with existing methods. On average, it showed 85.8% sensitivity and 78.2% specificity on simulated datasets. It also showed higher specificity and sensitivity using *n*-gram models built from reduced reference genomes and on models with lower coverage sequencing data. It outperforms alternative approaches in genus- and strain-level prediction and strain abundance estimation. Overall, the results show that StrainIQ achieves high accuracy by implementing customized model-building and is an efficient tool for site-specific microbial community profiling.

## 1. Introduction

Human microbiota form complex ecological communities that discretely inhabit various body parts. They play a critical role in human health and metabolism, where alterations in these microbial compositions could lead to various human diseases. The gastrointestinal (GI) tract and mouth are the largest ecological environments in the human body, with several distinct habitats supporting the dynamic growth of highly heterogeneous microbial species [1,2,3]. Most of the resident microbial communities in a healthy host contribute to various metabolic, physiological, and immune functions of the host. However, dysbiosis in the human microbiome (imbalance in composition and relative abundance of taxa) is associated with various human diseases or disorders [4]. Dysbiotic microbial communities influence the cellular processes of the host through altering the gut barrier functions and releasing bioactive metabolites and immune targets [5,6,7,8,9].

Hence, it is essential to understand the composition of microbial communities in human health and disease [10]. Extensive research on the human microbiome has shown its links with human pathologies, especially obesity [11,12], cancer [13], mental health issues [14], allergies, celiac disease, autism, type 2 diabetes mellitus, inflammatory bowel disease [11], gingivitis and periodontitis [3], which are associated with alterations (also referred to as dysbiosis) in microbial communities. The traditional approach limits microbiome research to exclusively study species that can be successfully cultured in the lab. Although some individual microbial species can have drastic effects on human health, it has now been identified that the microbial community plays a crucial role in the overall effect on the host’s health [11]. With the advent of next-generation sequencing technologies, our ability to identify microbial communities’ composition and function has increased rapidly. These technological advancements paved the way for the identification of several novel microbes, and have guided researchers to study the effects of human microbiota on various human diseases, such as inflammatory bowel disease [15], cancer [13], liver diseases [16], metabolic diseases [17], the effect of a mother’s microbiome on the infant’s microbiome [18], brain disease [19], infertility [20], gingivitis, periodontitis [21,22], and more. 

Traditionally, bacterial genomes have been reconstructed by sequencing deoxyribonucleic acid (DNA) from pure cultures and manually curating genomic contigs to generate high-quality drafts or complete genomes. But not all microbes can be cultured in the lab, owing to the inability to recreate their native growing conditions. Advancements in shotgun sequencing have led to the development of the culture-independent method of microbiome studies referred to as genome-resolved metagenomics. This has allowed the construction of whole genomes from environmental samples, generating a vast number of draft metagenome-assembled genomes (MAGs). While these methods resolve the limitations of the traditional approach, most of the MAGs are incomplete and suffer from assembly errors, gaps, chimeras, and contamination. Due to these limitations, close to 90% of bacterial genomes in the GenBank are currently incomplete [23].

Human microbiome studies have indicated that individuals tend to have a unique microbial composition, to the extent that they can act as microbial “fingerprints” [24]. Strain-level diversity is what uniquely identifies an individual’s microbiome. In many cases, strain-level variation also determines a microbe’s ability to cause diseases [25], resistance to antibacterial drugs [26], or be useful as precise markers to distinguish between human populations [3]. Hence, it is essential to identify microbes at a strain level to design an effective personalized treatment regimen for patients.

The identification and quantification of individual taxa in any metagenomics sample is highly dependent on the availability of high-quality reference genomes. Many tools have been developed to identify the taxonomic composition using short-read metagenomics data. Alignment-based methods such as MEGAN [27], MetaFlow [28], and PathoScope [29] infer the composition in each sample by aligning the reads to existing reference databases. Hence, these methods are highly dependent on the quality of the reference genomes. Other categories of tools used to analyze metagenomics data include *k*-mer-based methods such as Kraken [30], KrakenUniq [31], CLARK [32], CLARK-S [33], and LMAT [34]. These tools compare *k*-mers generated from the reads in metagenomics data against the reference genomes. Hence, the *k*-mer-based methods are relatively less sensitive to the quality of the reference genomes, as long-range alignments are not performed. Also, methods such as StrainEst [35] and ConStrains [36] use SNPs/SNVs and are highly dependent on the coverage. In the human body, microbial communities seem to be uniquely concentrated at different sites such as the gut, skin, and oral cavity [37]. The currently available methods, detailed by the authors in [27,28,31,32,38,39], primarily use more generic databases of reference genomes containing all the known microbial species from various body sites. This large search space can result in a significant number of false positives [28]. Hence, there is a need for a method that is developed around body-site-specific reference genome databases to obtain more accurate results. In this study, we proposed a novel *n*-gram-based method, StrainIQ, for the identification and quantification of microbial taxa at the strain level using whole-genome sequencing (WGS) metagenomic samples. StrainIQ takes advantage of the discriminative nature of unique *n*-grams as well as the weighted common *n*-grams present in incomplete and draft metagenomic assemblies. Additionally, StrainIQ leverages the body-site-specific reference genome information to increase the specificity of the prediction. In comparison to other metagenomic taxa profiling methods such as CLARK, MetaPhlAn, and KrakenUniq, our StrainIQ method showed superior performance using site-specific reference genome *n*-gram models. 

## 2. Materials and Methods

### 2.1. Site-Specific Reference Genome Sets 

A total of 2234 reference genome assemblies cataloged under the Human Microbiome Project (HMP) in the National Center for Biotechnology Information (NCBI) BioProject database were downloaded in September 2020. These genomes were sequenced under various sub-projects under HMP, and belong to different body sites, including the gastrointestinal (GI) tract, airways, oral cavity, skin, blood, and urogenital tracts. We downloaded the reference genome assemblies of the GI tract, blood, and urogenital tract and parsed those genome assemblies to remove the plasmids present in them. 

### 2.2. Building n-Gram-Based DNA Signature Element Models (DSEMs) 

**Figure 1:**
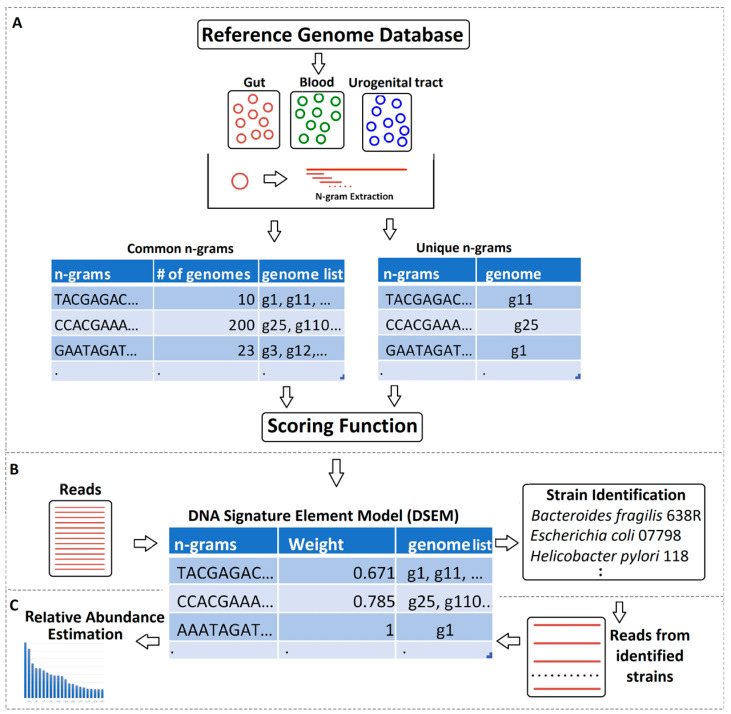
Graphical summary of StrainIQ algorithm. (**A**) *n*-gram quantification for DSEM building based on reference genomes. (**B**) Taxa identification and (**C**) relative abundance estimation of taxa from metagenomic data using DSEM. The longer red color lines in the figure indicate the linear genomes of the microbes from the reference genome and the shorter red lines denote the extracted *n*-grams.

We developed de novo *n*-gram reference genome models for each body site, called DNA signature element models (DSEMs), to predict and quantify body-site-specific taxa at various taxonomic levels. An *n*-gram is any contiguous sequence of DNA with a fixed length of *n* nucleotides. In computational genomics, *n*-grams are alternatively called *k*-mers or *n*-mers. The StrainIQ method uses unique (occurring in only one genome) and common (occurring in more than one genome) *n*-grams as signature elements for identifying taxa in metagenomic datasets. The de novo building of DSEMs includes the generation of *n*-grams from the reference genomes and scoring each *n*-gram using a scoring function, described in detail below. The methodology of building site-specific DSEMs is depicted in Figure 1. The score represents the discriminatory value of each *n*-gram in the site-specific genomes. An *n*-gram occurring in fewer genomes has a higher weightage (high discriminatory power) than those occurring in multiple genomes. 

#### 2.2.1. *n*-Gram Generation and Encoding

For building site-specific DSEMs, the reference genomes in corresponding body sites are disassembled to a list of contiguous *n*-grams. For a nucleotide sequence of length *x*, the generation of overlapping *n*-grams yields (*x − n* + 1)-many *n*-grams, where *n < x*. Only four bases (A, C, G, and T) are allowed in an *n*-gram; *n*-grams containing any other characters are ignored. In our DSEMs, *n*-grams were encoded using Huffman encoding [40] to increase efficiency and reduce memory and storage requirements. Then, the list of non-redundant *n*-grams was identified from the extracted *n*-gram for each genome and compared against those from other genomes in the body site to identify the unique and shared (common) list of *n*-grams for each body site. Also, we optimized the size of the *n*-gram by comparing the uniqueness of *n*-grams present in the reference genomes of a body site for different *n*-sizes. For this, we generated *n*-grams of sizes 12 through 27 with +3 increments (such as 12, 15, 18, and so on) and determined the common and unique *n*-grams for each case. As described in the results section, we found that *n* = 21 is the optimal size for use in DSEM building. Hence, we extracted unique and common *n*-grams of size 21 from all the genomes of each body site and indexed them to build site-specific DSEMs.

#### 2.2.2. Scoring Function

The purpose of the scoring function is to assign weights to the *n*-grams based on their discriminatory nature in the corresponding reference genome set. The unique *n*-grams are distinct to only one genome in a body site, and common *n*-grams occur in more than one genome. The scoring function considers the number of genomes an *n*-gram is present in and assigns an appropriate weight to the *n*-gram to reflect its discriminatory power. The scoring function implemented in this study was like the term “weighting”, as discussed in our previous study [41]. For any *n*-gram x, the score *S_x_* is given by the following expression:

$$S_{x}={\left(\ln\left(\frac{\left|c\right|}{\left|\left\{c:x\epsilon c\right\}\right|}\right)/\ln\left|c\right|\;\right)}^{2},$$

where *|c|* is the total number of reference genomes in the DSEM and |{*c: xϵc*}| is the total number of genomes in which *n*-gram *x* is present. 

For a unique *n*-gram,

$$\left|\left\{c:x\epsilon c\right\}\right|=1,\;$$

$$∵\;s_{x}=1.$$

The score for *n*-grams ranges between 0 and 1, where all unique *n*-grams will receive a score of 1, and those present in all the genomes will receive a score of 0. The square power in the denominator rapidly dampens the score for *n*-grams that are commonly present in multiple genomes; hence, *n*-grams that occur in fewer genomes receive a better discriminatory score closer to 1. A genome is predicted to be present in a sample based on the sum of the scores of all the *n*-grams; hence, *n*-grams with smaller scores can contribute to the decision-making process.

### 2.3. Identification of Strains from Metagenomic Sequencing Datasets

The identification step involves generating *n*-grams from the metagenomic sequencing reads and comparing them to DSEMs to identify a list of genomes in the metagenomic data. For this, we deconstructed the sequencing reads into unique overlapping *n*-grams and identified their scores based on the DSEM. For each metagenomic dataset generated for in silico and experimental validation, the reverse reads were converted to a forward direction and then all reads were combined to generate unique *n*-grams. We built a matrix *W* with genomes as columns and the *n*-grams as rows, and filled each cell in the matrix with the scores of the *n*-grams. For *N* = {*n*1, *n*22, *n*33 *…*, *nx*}, where *N* is a set of *n*-grams ranging from *n*1 to *nx*, generated from a metagenomic sequencing read, and *G* = {*g*1, *g*2, *g*3 *…*, *gy*}, where *G* is a set of reference genomes ranging from *g*1 to *gy* in a body site, we have

$$W=(w_{nxgy})=\left[\begin{array}{c} w_{n1g1\;}\\ w_{n2g1\;}\\ \begin{array}{c} \vdots \\ w_{nxg1\;} \end{array} \end{array}\;\;\;\;\begin{array}{ccc} w_{n1g2\;} & \cdots & w_{n1gy\;}\\ w_{n2g2\;} & \cdots & w_{n2gy\;}\\ \begin{array}{c} \vdots \\ w_{nxg2\;} \end{array} & \begin{array}{c} \ddots \\ \cdots \end{array} & \begin{array}{c} \vdots \\ w_{nxgy\;} \end{array} \end{array}\right].\;$$

The summed column score (*gj*-th column) in the matrix represents the score ($S_{gj}$) for an individual genome and that gives the initial probability of the presence of a specific taxon in the metagenomic sample:

$$S_{gj}=\overset{x}{\underset{ni=1}{\sum }}w_{nigj},\;$$

where *x* is the total number of *n*-grams in the metagenome and *gj* is the *gi*-th genome in the matrix. We calculated the sum for each column in the matrix, represented below:

$$S={\left(S_{gj}\right)}_{gj=1}^{n},$$

where *S* is a vector of column scores and *n* is total columns/genomes in the matrix. Then, each column score *S_gj_* is further normalized using a parameter (*nFactor_g_*) that considers the size of the genome and the number of *n*-grams contributing to the prediction score. The *nFactor_g_* is defined as

nFacorg=ncnt ,

where *n_c_* is the number of *n*-grams that contributes to the score *Sgi* and *n_t_* is the total number of *n*-grams in the genome. The final probability score (*fS_gi_*) is calculated as below:

$$fS_{gi}=S_{gi}*\;nFactor_{g}$$

The *fS_gi_* values are further processed based on the cutoff scores to identify the presence of a genome in the metagenomic community. While our methodology is generic and works with all body sites, we used the gastrointestinal (GI) tract microbiota for testing purposes in this study as they contain the highest number and most diverse set of taxa. 

We calculated the cutoff scores to avoid the prediction of any random genome that can be predicted simply by chance because of the common *n*-grams present within the genomes of a body site. Note that these cutoffs should be determined for each body site separately as they vary for each body site depending on the number and composition of the genomes in that microbiome. Here, we showed the example of the GI tract microbiota. To determine the optimal score cutoff, we used these scores’ distributions from positive and negative datasets described in our previous study [42]. For example, to estimate the cutoff score for GI tract strain identification, we simulated a metagenome containing 471 reference genomes from the GI tract as the positive dataset and selected the same number of non-GI tract genomes as the negative dataset. We calculated the prediction scores for each genome in the positive and negative datasets using the GI-tract-specific DSEM and prediction algorithm. Then, we plotted the score distributions of the genomes from the GI tract in descending order and those from the negative dataset in ascending order. The intersection of these two plots is deemed to be the score cutoff, where the ascending score of the negative dataset exceeds the descending score of the positive dataset. In other words, at this cutoff, the method predicts maximum true positives and minimum false positives.

### 2.4. Quantification of Strains

Relative abundance is calculated by assigning the metagenomic reads to identified genomes based on the unique and common *n*-grams present in the reads. With *n* = 21, we identified that a significant number of *n*-grams were unique to single genomes. The distribution of unique and common *n*-grams of the GI tract reference genomes is provided in Supplementary Table S6. We calculated the read-genome score for reads containing only non-unique *n*-grams to assign those reads to appropriate genomes. The read-genome score for a read is the sum of the weights of the *n*-grams that are common between a genome and a read. For *N =* {*n*1, *n*2, *n*3, *…*, *nx*}, where *N* is a set of *n*-grams present in reads *R =* {*R*1, *R*2, …, *Rj*} containing only non-unique *n*-grams, the read-genome score (*R_jg_*), i.e., the probability of *Rj* belonging to a specific genome, is calculated as

$$R_{jg}=\overset{x}{\underset{ni=1}{\sum }}S_{nigy},$$

where *x* is the total number of *n*-grams in a metagenomic read and *gy* is the total number of genomes in a body site. The read is assigned to a genome with a maximum *R_jg_* score. 

### 2.5. In Silico and Experimental Validation Using Simulated and Mock Communities

#### 2.5.1. Simulated Datasets for Testing

We used InSilicoSeq [43] software with the NovaSeq error model to simulate ten metagenomes with 20 million 150 bp paired-end reads from 200–300 randomly selected reference genomes from the GI tract (Supplementary Table S3, Supplementary Dataset 2). The simulated reads from InSilicoSeq are similar to reads from Illumina sequencing. It provides a flag to use draft genomes for simulation; this enabled us to use all draft genomes for creating test samples. 

#### 2.5.2. Mock Community Datasets for Testing

We also used the Gut Microbiome Genomic Mix (ATCC^®^ MSA-1006™) containing 12 evenly mixed gut genomes and Staggered Mix Genomic Material (ATCC^®^ MSA-1003) containing 20 staggered mix genomes from ATCC (https://www.atcc.org/, accessed on 4 January 2021) for experimental validation. Since most of the strains included in these mock samples are complete and not present in the NCBI databases, we obtained the complete genomes from ATCC and updated our DSEM before testing. As most of the reference genomes available at NCBI are drafts, we performed additional tests with only 75%, 50%, and 25% of the ATCC reference genomes to explore the robustness of the StrainIQ by recreating DSEMs with partial genomes. In addition, we also reduced the sequencing data size for the mock communities to simulate lower sequencing coverage of the genomes and tested the strength of the StrainIQ. The initial sequencing produced reads at approximately 120x coverage for 12 genomes in each sample set (Supplementary Datasets 3 and 4). From this, we generated additional test sets representing 90x, 60x, 30x, 5x, 3x, and 1x coverage of the genomes by sampling only a subset of the sequencing reads. The coverage was calculated based on the size of the genomes in the mock community.

### 2.6. Comparison against Other Popular Methods

We compared StrainIQ to other popular methods including KrakenUniq [31], CLARK [32], and MetaPhlAn [39]. For KrakenUniq and CLARK, we created a customized database with GI tract reference genome assemblies from HMP NCBI BioProject to match the reference genomes used by StrainIQ, and MetaPhlAn was implemented with its default database. We ran each of these methods to identify the genomes present in the ten simulated metagenomes and compared the results against StrainIQ at different taxonomic levels. Further, we ran strain-level identification and quantification using StrainIQ and KrakenUniq (with default settings) on both simulated and experimental datasets, and compared the performance of StrainIQ against KrakenUniq. 

### 2.7. Statistical Measures Used for Performance Testing 

We used sensitivity, specificity, and F1 score to evaluate the performance of StrainIQ to predict the taxa present in the metagenomic samples. The values of these measures range from 0 to 1, where 1 indicates the best prediction accuracy and vice versa. Each measure is described in the context of the StrainIQ validation below:

Sensitivity/true positive rate/recall: This refers to the StrainIQ’s ability to correctly identify the microbes present in the sample.

$$Sensitivity=\frac{TP}{TP+FN}\;.$$

Specificity/true negative rate: This refers to the StrainIQ’s ability to correctly identify the microbes that are not present in the sample.

$$Specificity=\frac{TN}{TN+FP}\;.$$

F1 score/F-measure: The F1 score is calculated from the harmonic mean of precision and recall. It measures the StrainIQ’s overall accuracy, which makes it ideal for the cases where sensitivity and specificity are not enough to correctly distinguish the merits of the methods.

$$F1\;Score=\frac{TP}{TP+\frac{1}{2}\left(FP+FN\right)}\;.$$

In the above equations, we use the following terms:

TP—True positive. The number of microbes correctly identified as being present in a sample; 

TN—True negative. The number of microbes correctly identified as not being present in a sample;

FP/type I error—The number of microbes incorrectly identified as being present in a sample;

FN/type II error—The number of microbes incorrectly identified as not being present in a sample.

## 3. Results

### 3.1. n-Gram-Based Body-Site-Specific DSEMs

Our novel StrainIQ algorithm identifies the taxa at different levels including strains from metagenomics samples. As different microbial floras inhabit different body sites, we resort to developing body-site-specific DSEMs to enable accurate prediction. In this study, we built site-specific DSEMs for the GI tract, blood, and urogenital tract using 488, 54, and 359 genomes, respectively. For the GI tract, we used 459 draft and complete genomes from NCBI and 29 mostly complete genomes from atcc.org mock communities (ATCC^®^ MSA-1006™, ATCC^®^ MSA-1003™), while the genomes inhabiting the blood and urogenital tract were downloaded from NCBI. Overall, we built separate DSEMs for each body site that has at least 50 identified genomes to confer enough discriminatory power to the model. The DSEMs can be built using this method for any other body site containing at least 50 genomes. In this study, we described our model using GI tract DSEM implementation and testing procedures.

### 3.2. Identification of Optimal Size of an n-Gram for DSEM Building

Determining the optimal size of *n*-grams is an important step for the optimal performance of the StrainIQ algorithm. We optimized the size of the *n*-gram based on two factors: the number of unique *n*-grams and the total number of *n*-grams in the DSEM. A large *n*-gram size increases the discriminatory power at the expense of the memory and processing time for the tool. In contrast, a small *n*-gram size involves the risk of losing the discriminatory power to identify strain-level differences in a sample. Hence, to determine the optimal *n*-gram size, we generated *n*-grams for *n* = 12, 15, 18, 21, 24, and 27 from all the body-site-specific reference genomes and identified the unique and common *n*-grams. We tried different n values as multiples of three because the genetic code is a triplet code made of a series of three nucleotides [44]. Supplementary Table S1 shows the number of unique/common *n*-grams, and the memory and time required for generating the *n*-grams using the GI tract reference genomes. The number of unique/common *n*-grams increases with the size of n. For *n* = 12, the number of unique *n*-grams is only 14,933, whereas for a higher n, the number of unique *n*-grams is in the millions (for *n* = 27, the number of unique *n*-grams is 837,006,517). We observed that there was not much of a further increase in the numbers of unique *n*-grams as the *n*-gram size increased beyond 21. However, there was a significant increase in the memory requirement and the *n*-gram generation time with increases in the size of n (Supplementary Figure S1). Hence, we chose 21 as the optimal size for n and used this for generating models for the GI tract and other body sites. 

### 3.3. DSEM Building from GI Tract Reference Genomes

We built a 21-g DSEM for the GI tract using 488 microbial genomes that inhabit the GI tract (including mock community microbes), which contained a total of 988,866,457 *n*-grams. Of these, 809,679,392 (~81%) were unique to individual genomes in the set and the rest were shared by multiple genomes. The number of unique *n*-grams ranged from 85 to 9,144,371 in different genomes (Supplementary Table S5). The higher the number of unique *n*-grams, the more distinct the species is with respect to the other genomes in the set. Each *n*-gram is assigned a score that ranges from 0 to 1. The scoring function was designed to assign the full weight to unique *n*-grams and a rapidly decaying weight to the common *n*-grams as they become more common in the genome set (Supplementary Table S2). We also created DSEMs for the blood, which has 54 genomes, and the urogenital tract, with 359 genomes.

### 3.4. Threshold Score Cutoff for Taxa Prediction

**Figure 2:**
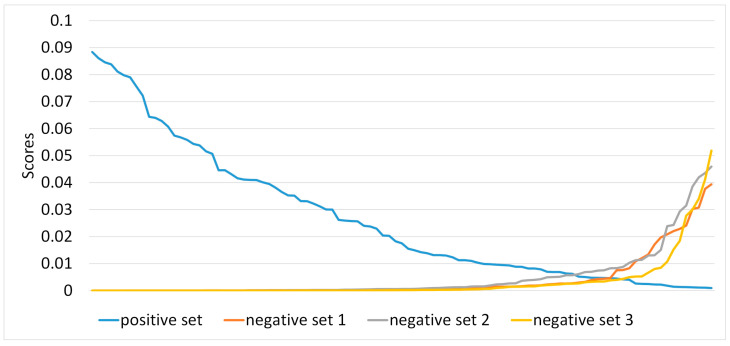
Determining the *n*-gram score cutoff for the GI tract DSEM. The intersection point between the positive and negative datasets is the optimal cutoff where there will be maximum true positives with minimum false positives.

To avoid the false positive prediction of site-specific genomes, we estimated the threshold cutoff scores for each body site by plotting the genome prediction score distribution of positive and negative datasets (Supplementary Dataset 1). We determined the optimal cutoff to be 3.16 × 10^−9^ (Figure 2) based on our de novo built GI tract DSEM. The point at which the positive and negative scores intersect is the optimal cutoff where there are maximum true positives with minimum false positives. Similarly, we calculated the cutoffs for the blood and urogenital tract using corresponding DSEMs and appropriate positive and negative datasets (Supplementary Table S9). In an ideal case, we expect the maximum prediction score for any genome in the negative datasets to be less than the minimum score of the genomes in the positive datasets. However, plenty of *n*-grams of size 21 can occur in both negative and positive datasets, resulting in cases where the genomes in the negative datasets have significant scores and exceed those of the genomes in the positive dataset. Figure 2 shows the scores for the positive and negative datasets and the intersection point for the GI tract. The prediction score distribution in the negative datasets before the intersection is represented by *n*-grams that are less discriminatory, and those beyond the intersection point are more discriminatory than those of the positive dataset. In other words, the intersection is the score threshold where *n*-grams from the positive dataset have higher discriminatory power than those in the negative dataset to identify the taxa accurately. The values beyond the intersection indicate the scores that any random genome can have because of the common *n*-grams. We considered the intersecting point a threshold cutoff score to distinguish positive genomes in the metagenomic dataset and avoid any other random genome match in the DSEM-based prediction model.

### 3.5. Assessing the Performance of the StrainIQ Algorithm Based on Simulated Datasets

**Figure 3:**
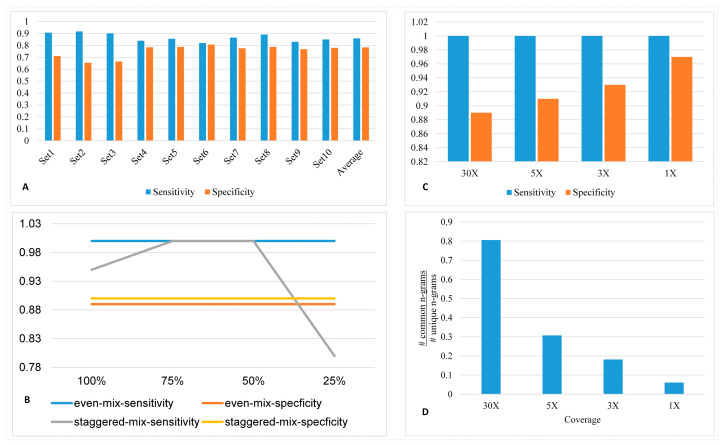
StrainIQ results for simulated and experimental samples. Sensitivity and specificity plot (values on y-axis) (**A**) using ten simulated datasets; (**B**) using even and staggered experimental samples across different reference quality (on x-axis); and (**C**) using different coverages (on x-axis). (**D**) Comparison of uniqueness of *n*-grams across different coverages. The y-axis is the ratio of the number of common *n*-grams in the group to the number of unique *n*-grams. # mark in figure indicates ‘number of’.

We assessed the accuracy of the StrainIQ algorithm using 10 stimulated metagenomic datasets with the known composition of microbial genomes. The genomes were selected randomly from GI tract reference genomes to build simulated metagenomic datasets. The details of the simulated sets are shown in Supplementary Table S3. Set 1, Set 2, and Set 3 were simulated using 300 genomes from the GI tract and Sets 4 through 10 were simulated using 200 genomes from the GI tract. Then, we tested StrainIQ against these datasets to evaluate its performance. Our method was able to identify taxa at the strain level in the simulated datasets at an average of 0.858 sensitivity and 0.782 specificity (Figure 3A) We noticed that the specificity for the datasets containing a larger number of genomes (Sets 1–3) was lower and the sensitivity was higher compared to corresponding values for the datasets containing fewer genomes.

### 3.6. Assessing the Performance of the StrainIQ Algorithm on Experimental Datasets

We sequenced mock communities containing an even and staggered mix of genomes from atcc.org (ATCC^®^ MSA-1006™, ATCC^®^ MSA-1003™). This allowed us to validate the strength of the tool with known standards of mock communities. Moreover, using the staggered mix, we explored the strength of the tool to identify the less abundant genomes in the sample, as it contains the microbial genomes with varying compositions from 0.02% to 18.0%. The mock communities were sequenced on the NextSeq550 to generate 150 bp paired-end reads. The sequencing details can be found in Supplementary Table S4. To test these mock communities, we custom-built separate GI tract DSEMs by including 100%, 75%, 50%, and 25% of the mock community genomes. Supplementary Tables S5 and S6 show the *n*-gram statistics before and after adding the new mock community genomes in DSEM. These data show that the addition of the new genomes reduced the number of unique *n*-grams in the DSEM (Supplementary Figure S2). Figure 3B shows the performance of StrainIQ for even and staggered mix mock communities against the four custom-built DSEMs. We observed a specificity of approximately 0.88 for both even and staggered communities and 1.00 sensitivity for the even community. For the staggered community, the sensitivity dropped to 0.79 for the model with only 25% of the reference genomes, while for the other three DSEMs at 50%, 75% and 100% reference genomes, the sensitivity exceeded 0.93. The sensitivity and specificity measures were constant across DSEMs with different proportions of reference genomes for the even communities, whereas for staggered communities, the sensitivity and specificity measures varied across DSEMs built with different proportions of reference genomes. Overall, StrainIQ showed a reasonably high level of sensitivity and specificity despite using reduced reference genome models for strain-level prediction. This proves the strength of our algorithm to accurately identify strains in a metagenomics sample even when the reference assemblies are incomplete and at different stages of draft genomes. We also noted that the identification algorithm could accurately identify strains with similar sensitivity and specificity for both even and staggered mixed samples. 

The microbiota often shows a huge variation in the relative abundance of its constituent taxa. The highly abundant taxa represent the core of the metagenomic community, and the rare taxa represent a small fraction of the metagenome community. Hence, in this study, we tested StrainIQ’s performance using datasets with different sequencing coverages. We created four datasets to represent only 1x, 3x, 5x, and 30x coverage, as explained in the Methods section, and predicted the strain-level taxa in those datasets using the GI-tract-specific DSEM. Figure 3C shows the sensitivity and specificity at different levels of coverage. This analysis showed the same level of sensitivity (1.00) for all four datasets with different levels of coverage. However, the specificity increased from 0.89 to 0.97 as the coverage of the data decreased. This might be due to decreased false positives from decreased repeating common *n*-grams at lower coverages. As the coverage of sequencing data decreases, the percentage of repeating *n*-grams gradually decreases, and this improves the specificity at lower coverages. Although not seen in this case, this can also reduce the sensitivity when the genomes present in the samples rely mostly on common *n*-grams for identification. We analyzed the *n*-grams representing the different coverages of sequencing data to calculate the ratio of common *n*-grams to unique *n*-grams (Figure 3D). As expected, we observed that the number of unique *n*-grams increased from 5x coverage to 1x coverage compared to common *n*-grams. This is reflected in the specificity increase shown in Figure 3C. 

### 3.7. Comparison of StrainIQ Performance with other Popular Methods

We compared the performance of StrainIQ against three other popular tools used for metagenomics analysis, namely, KrakenUniq, MetaPhlAn, and CLARK, using sensitivity and specificity metrics. Supplementary Table S8 shows the comparison of sensitivity and specificity of all four methods at the species, genus, and strain levels. In this study, we presented the F1 measure for comparison, which signifies the accuracy of a method by combining the precision and recall (sensitivity). Table 1 lists F1 score for different methods at the strain, species, and genus levels. 

For this comparison, we chose the GI tract body site and used three sets of simulated genomes from the GI tract to calculate the average F1 for each method. The StrainIQ performance was superior to both MetaPhlAn and CLARK, and at the genus level, had an F1 score of 0.977. In comparison, the F1 score of MetaPhlAn and CLARK at the genus level were 0.914 and 0.887%, respectively. Also, StrainIQ performed better than CLARK and MetaPhlAn at species-level prediction, while the results at the strain level could not be compared, as the latter methods do not predict at the strain level (Table 1). The CLARK method has shown a very high number of false positives compared to other methods, resulting in a very low specificity of a mere 3.5% (Supplementary Table S8). StrainIQ has better specificity than CLARK and is more sensitive than MetaPhlan. On the other hand, KrakenUniq slightly outperforms StrainIQ at the genus level, and significantly at the species level, with F1 score of 0.983 and 0.942, respectively. However, StrainIQ outperforms at the strain level with an F1 score of 0.821 in comparison to only 0.639 for KrakenUniq.

**Figure 4:**
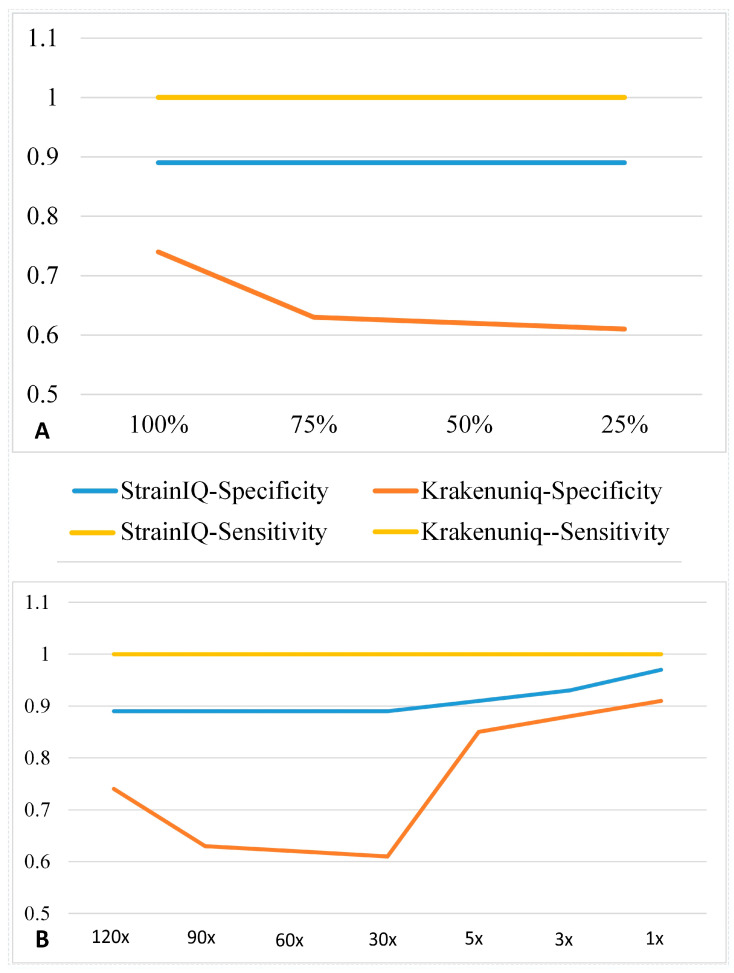
Comparison of sensitivity and specificity between StrainIQ and KrakenUniq in strain-level identification using (**A**) complete and incomplete reference genome models and (**B**) different metagenomic sequencing coverage datasets. Note that the yellow lines shown in both Figure 4A,B represent the sensitivity measures for both StrainIQ and KrakenUniq as those values are identical at 1.

We also used mock microbial communities to compare StrainIQ against KrakenUniq at the strain level. To investigate the effects of incomplete reference genome models (DSEMs), we ran both StrainIQ and KrakenUniq against four custom-built DSEMs (100%, 75%, 50%, and 25% of genomes). Figure 4A shows the comparison of specificity and sensitivity between the two methods against the four models. The sensitivities of the two methods were remarkably identical at 100% (with the two lines merged as one) without any effect of the incompleteness of the models. StrainIQ showed the same level of specificity at around 90% for all models, while KrakenUniq showed a big drop from approximately 0.75 with the 100% genome model to 0.61 with the 25% genome model. These results demonstrate that the StrainIQ algorithm is very robust and performs consistently better than KrakenUniq, even with incomplete draft genomes for strain-level identification.

We also assessed the performances of StrainIQ and KrakenUniq at different sequencing coverages of the mock sequencing dataset ranging from 120x to 1x coverage using the sensitivity and specificity measures based on the taxa identification (Figure 4B). We found that the sensitivity remained consistent for both StrainIQ and KrakenUniq with the highest value of 1.00, while StrainIQ showed consistently higher specificity (from 0.89 to 0.97) than KrakenUniq (from 0.61 to 0.91) at all the sequencing coverages tested. However, both StrainIQ and KrakenUniq showed increased specificity as the sequencing coverage decreased from 30x to 1x, especially at the lower sequencing coverages from 5x and 1x. 

### 3.8. Quantification of the Identified Taxa from the Metagenomic Data 

The StrainIQ algorithm estimates (Figure 1C) the relative abundance of the microbes that are present in the metagenomic sequencing datasets by assigning the reads to corresponding taxa identified in the first step (Figure 1B). In this study, we tested the performance of StrainIQ quantification using simulated datasets and sequencing reads from experimental mock communities in comparison to KrakenUniq. We generated ten simulated datasets with known relative abundances for each taxon in these datasets and used them to test the quantification performance of StrainIQ and KrakenUniq. Based on the difference between the predicted and simulated relative abundance values, we determined the number of genomes each method predicted better, i.e., closer to the known values, for all ten sets. Table 2 lists the number of genomes predicted by StrainIQ and KrakenUniq and the differences in predicting true positives. The first column shows the ten datasets tested. The second and third columns include the number of all genomes predicted by each method (including false positives). The last column “StrainIQ’s lead (%)” shows the percentage difference in predicting the true positive genomes by StrainIQ in comparison to KrakenUniq. Even though both methods showed false positives, StrainIQ’s quantification performance of relative abundance was much better than that of KrakenUniq, while KrakenUniq performed slightly better with datasets 3 and 6. StrainIQ outperformed in 8 out of 10 datasets, and was able to quantify closer to the known values for higher number of genomes (from 3% to 37%) over KrakenUniq.

**Figure 5:**
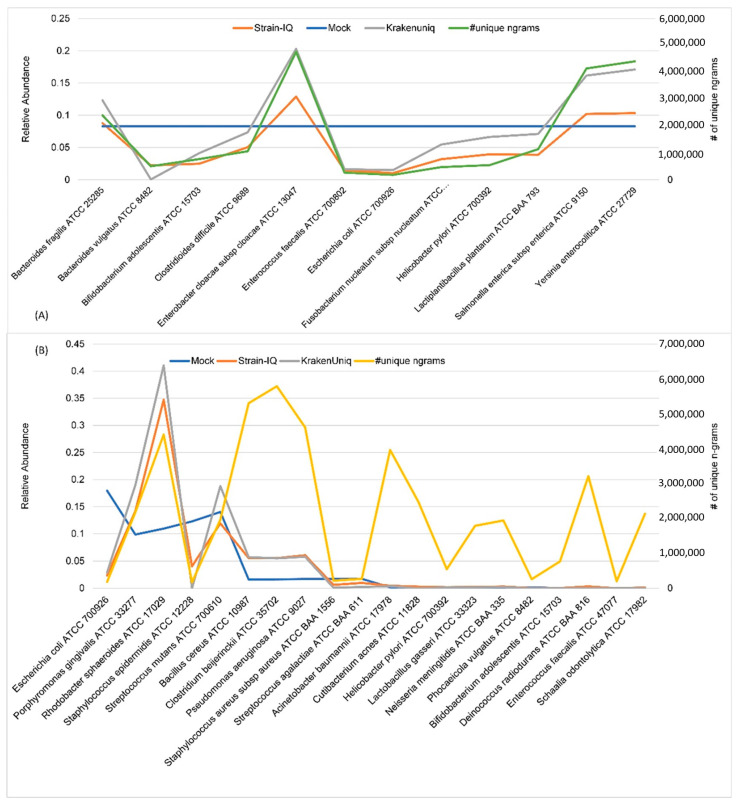
Comparison of relative abundance estimates of sequenced mock communities (with known relative abundances) using StrainIQ and KrakenUniq, in proportion to the number of unique *n*-grams in (**A**) even community and (**B**) staggered community. The line added parallel to the y-axis (relative abundance) on right of the graph represents the number of unique *n*-grams in each genome in the even (green line) and staggered (orange line) mix samples. # symbol in the figure indicates ‘number of’.

Among the mock experimental samples, even communities have 12 genomes with an even relative abundance of 0.083 each, and the staggered communities have 20 genomes with varying relative abundances ranging from 0.0002 to 0.18. The actual relative abundances of taxa in these communities are shown in Supplementary Table S7. The estimated relative abundances of both even and staggered mock communities using StrainIQ and KrakenUniq are shown in Figure 5. This analysis showed a similar performance of StrainIQ and KrakenUniq in estimating the even and staggered mock community species. However, it also showed a higher number of false positives in these two small communities, with 162 and 56 false positives in the even community by KrakenUniq and StrainIQ, and 89 and 49 false positives in the staggered community by KrakenUniq and StrainIQ, respectively (not shown in Figure 5). We compared each identified microbe’s relative abundance to the corresponding number of its unique *n*-grams in the even and staggered communities for both the StrainIQ and KrakenUniq tools. Both KrakenUniq and StrainIQ showed an overestimation of Enterobacter cloacae subsp. cloacae ATCC 13047 and an underestimation of Escherichia coli ATCC 700926 in association with their numbers of unique *n*-grams at 4,763,541 and 180,987, respectively, in the even community. Similarly, Rhodobacter sphaeroides ATCC 17029 was overestimated and Staphylococcus epidermidis ATCC 12228 was underestimated in the staggered community, and the estimates were in proportion to their numbers of unique *n*-grams, at 4,403,102 and 173,174, respectively. Both StrainIQ and KrakenUniq overestimated the taxa with higher number of unique *n*-grams and underestimated the taxa with a smaller number of unique *n*-grams. However, these analyses revealed that the accuracy of the relative abundance estimation of microbes in a microbial community depends upon the number of unique *n*-grams identified in each microbe.

## 4. Discussion 

Over the past several years, many tools have been developed for the taxonomic profiling of microbial communities, including reference-based alignment [27,28,29], marker-gene-based identification [39,45], and *k*-mer-based alignment-free methods [30,31,32,33]. The reference-based taxonomic profiling tools show higher accuracy, but run slower as the volume of the metagenomic reads in datasets increases, whereas marker-gene-based tools depend on the curated reference databases. In contrast, alignment-free methods are faster but need high-coverage sequencing data and reference genome sequences of all known microbes. Contrary to existing alignment-free methods, our de novo StrainIQ method leverages discrete small-number site-specific reference genomes to predict site-specific genomes more accurately from metagenomic datasets. We also employed the Huffman encoding method to encode binary *n*-grams and optimized the *n*-gram size to reduce the memory requirements significantly. Unlike most other methods, we use a comprehensive list of all overlapping *n*-grams for building DSEMs and taxa prediction, which requires us to store and process large amounts of *n*-gram data. StrainIQ uses the discriminatory nature of unique and weighted common *n*-grams to identify the taxa in any metagenomic samples. The *n*-grams occurring in fewer genomes are assigned higher weights, and the weights for *n*-grams decay rapidly as their frequency of occurrence increases. This scoring method allows us to reward the more discriminatory *n*-grams, while utilizing the weights of all the *n*-grams in the set.

With the appropriate size of *n*, the combination of unique and weighted common *n*-grams can distinguish taxa present in any metagenomic samples with high accuracy. The *n*-gram size is a critical factor to yield unique *n*-grams and manage memory size. We optimized the *n*-gram size and chose *n* = 21 for building body-site-specific DSEMs. At this *n*-gram size of 21, we found more unique *n*-grams for each body-site-specific genome and less memory to store *n*-grams when compared to an *n*-gram size of more than 21. 

Our method uses the knowledge of body-site-specific microbial communities to accurately identify and quantify the genomes. The DSEMs are built for each body site based on the genomes of microbes known to reside in the body site. This helps to reduce false positives significantly. The tool is easily customized for other environments such as ocean floors, ponds, and agricultural sites for accurate identification and quantification by building the environment-specific DSEMs. 

StrainIQ makes use of complete overlapping *n*-grams from the reference and input samples, allowing it to accurately identify the strain-level taxa at a higher resolution. Unlike KrakenUniq, which uses the classification of Kraken [18] at higher resolution (species) to derive strain identification, StrainIQ focuses initially on identifying strains and builds upwards to calculate higher taxa, making it more accurate for strain-level predictions. Our method showed a better performance than other metagenomic prediction tools such as CLARK, MetaPhlAn, and KrakenUniq at the strain level. This is due to its algorithm, which builds comprehensive reference genome-based models for body-site-specific data and objectively utilizes the de novo identified signatures (*n*-grams) in the metagenomic sequencing data without the need for pre-curated reference signatures. Its performance in higher-level taxa prediction is better than that of MetaPhlAn or CLARK, but not as good as KrakenUniq. The present version of the StrainIQ algorithm was implemented to capture the uniqueness of *n*-grams at the strain level; hence, it shows a higher level of performance for strain-level predictions than for higher taxonomic levels. The relative abundance predicted by StrainIQ and KrakenUniq follows a similar trend, as shown in the relative abundance estimation of simulated and mock communities (Figure 4 and Figure 5), but StrainIQ has a lower false positive rate compared to KrakenUniq. Even so, the relative abundance estimation of both methods depends on the number of unique *n*-grams in the reference genomes; the higher specificity shown by StrainIQ might be attributed to the site-specific DSEMs implemented in the algorithm. Our abundance analysis showed that the number of unique *n*-grams identified in each microbe influences the accuracy of the relative abundance estimation, which in turn, depends upon the optimal size of the *n*-gram and the number of reference genomes used for prediction. 

The analysis of StrainIQ prediction on simulated datasets with GI tract genomes revealed that the specificity decreases as the diversity of the community increases (Sets 1–3) and sensitivity increases as the diversity of the community decreases (Sets 4–10) (Figure 3). We presume that the lower specificity for highly diverse communities is due to the lower number of unique *n*-grams per genome, and the higher sensitivity for less diverse communities is due to the higher proportion of common *n*-grams.

The StrainIQ method can be easily implemented in other environments with minor modifications; the only requirement is the availability of site-specific reference genome assemblies, which are available from NCBI and other public genome databases such as the Joint Genome Institute’s (JGI) Genomes Online Database (GOLD) [46]. In addition to the GI tract, we built DSEMs for the blood and urogenital tract, and the cutoff scores and performances of those models are provided in Supplementary Table S9. The major advantages of StrainIQ over other methods include: there is no requirement for a pre-curated set of microbial DNA signatures; incomplete reference/draft genomes are sufficient to build the DSEMs as the algorithm makes use of both unique and common *n*-grams; metagenomic sequencing reads with lower or inconsistent coverage can be efficiently used for taxa identification and quantification. The weaker performance of StrainIQ in predicting higher-level taxa is attributed to the lack of separate DSEMs built at those taxonomic levels. This can be addressed in future versions of the method, because this method is mainly focused on strain-level identification. In the present study, we were able to use only a small set of mock community genomes for validations; hence, these results may not be generalized in comparison with other methods. In addition, StrainIQ demands cutoff score calculations for each set of site-specific community profiling because the composition and distribution of unique *n*-grams vary in each site-specific microbiome.

## 5. Conclusions

We developed an *n*-gram-based algorithm, StrainIQ, which builds de novo *n*-gram models utilizing total unique and common *n*-gram features of the site-specific genomes upon weighted scoring to predict and quantify the taxa present in whole-genome metagenomic samples. Additionally, StrainIQ employs the Huffman encoding of *n*-grams for memory and runtime management. This is followed by the optimization of *n*-gram size and the prediction of cutoff scores to improve the sensitivity and specificity of the prediction and quantification of taxa in metagenomic samples. StrainIQ showed an average of 0.858 sensitivity and 0.782 specificity on 10 simulated datasets with varying compositions. Furthermore, it showed high performance for mock communities utilizing incomplete reference genome models, and had a varying range of sequencing coverage of metagenomic samples. In comparison to other methods such as CLARK, MetaPhlAn, and KrakenUniq, StrainIQ showed superior performance both in terms of prediction and abundance estimation at the strain level. Finally, this method is highly adaptable to customize and train the microbiota of a particular body site or an environment. The software tool allows the users to identify the strains and higher-level taxa present in any metagenomic samples if the models are custom built using the reference genomes of species corresponding to that microbiome. The software tool is freely available and platform-independent, and it can be downloaded and utilized by any user with a basic computational background and programming experience.

## Figures and Tables

**Table 1:** Comparison of F1 score between StrainIQ, KrakenUniq, MetaPhlAn, and CLARK at different taxonomic levels.

	Genus	Species	Strain
StrainIQ	0.977	0.886	0.821
KrakenUniq	0.983	0.942	0.639
MetaPhlAn	0.914	0.719	NA
CLARK	0.887	0.719	NA

**Table 2:** Comparison of relative abundance prediction between StrainIQ and KrakenUniq.

Sets	StrainIQ	KrakenUniq	StrainIQ’s Lead (%)
Set 1	211	176	11.67
Set 2	196	190	2.00
Set 3	190	198	−2.67
Set 4	183	140	21.50
Set 5	175	143	16.00
Set 6	147	151	−2.00
Set 7	203	127	38.00
Set 8	187	145	21.00
Set 9	179	147	16.00
Set 10	173	142	15.50

## Data Availability

The datasets used in this study are available at https://zenodo.org/record/8132164, accessed on 11 July 2023. Figure2-datasets.zip file contains the simulated positive (from GI tract genomes) and negative (from non-GI tract genomes) metagenomic datasets; Simulated_datasets.zip file contains the simulated metagenomic sequencing data (from GI tract reference genomes); Gut_even.zip file contains the metagenomic sequencing data (~120X coverage) of the ATCC Gut Microbiome Genomic Mix (MSA-1006); and Gut_mix.zip file contains the metagenomic sequencing data (~120X coverage) of the ATCC 20 strain staggered genomic mix (MSA-1003).

## Supplementary Materials

The following supporting information can be downloaded at: https://www.mdpi.com/article/10.3390/genes14081647/s1, Table S1: Number of unique/common *n*-grams, and the memory and time requirements for the *n*-gram generation from the GI tract reference genomes; Table S2: Examples of weights assigned to unique and common *n*-grams based on the scoring function used while building DSEMs from the GI tract genomes; Table S3: Details of the simulated datasets, set 1 to set 10 built from the GI tract genomes; Table S4: The number of sequencing reads and *n*-grams generated for the mock communities used in this study; Table S5: Genome-wide distribution of non-repetitive and species-specific *n*-grams generated from 488 GI tract microbes and the mock community microbes (used for validation); Table S6: The distribution of unique and common *n*-grams of the GI tract reference genomes (N = 459); Table S7: Relative abundances of taxa in the mock (mix/staggered and gut/even) communities; Table S8: Performance comparison of StrainIQ against KrakenUniq, MetaPhlAn, and CLARK methods at the species, genus, and strain levels; Table S9: Description of the cutoff and performance scores of bodysite-specific DSEMs built using corresponding reference genomes; Figure S1: Correlation between the *n*-gram size and number of unique/common *n*-grams, memory requirement, and runtime using the GI tract reference genomes; Figure S2: Visualization of the percentage of unique *n*-grams in DSEMs with only GI tract genomes (GI DSEM), and with GI tract and mock community genomes (GI Mock DSEM.

## Abbreviations

DNA	deoxyribonucleic acid
DSEM	DNA signature element model
GI tract	gastrointestinal tract
HMP	human microbiome project
MAG	metagenome-assembled genomes
NCBI	National center for biotechnology information
