# Supplementary material for: StrainIQ: A Novel n-Gram-Based Method for Taxonomic Profiling of Human Microbiota at the Strain Level
Source: Genes (Basel). 2023 Aug 18;14(8):1647. doi: 10.3390/genes14081647 (PMC10454763; doi:10.3390/genes14081647)
Supplement: Supplementary file 1 [file genes-14-01647-s001.zip › Supplementary Figures.pdf]

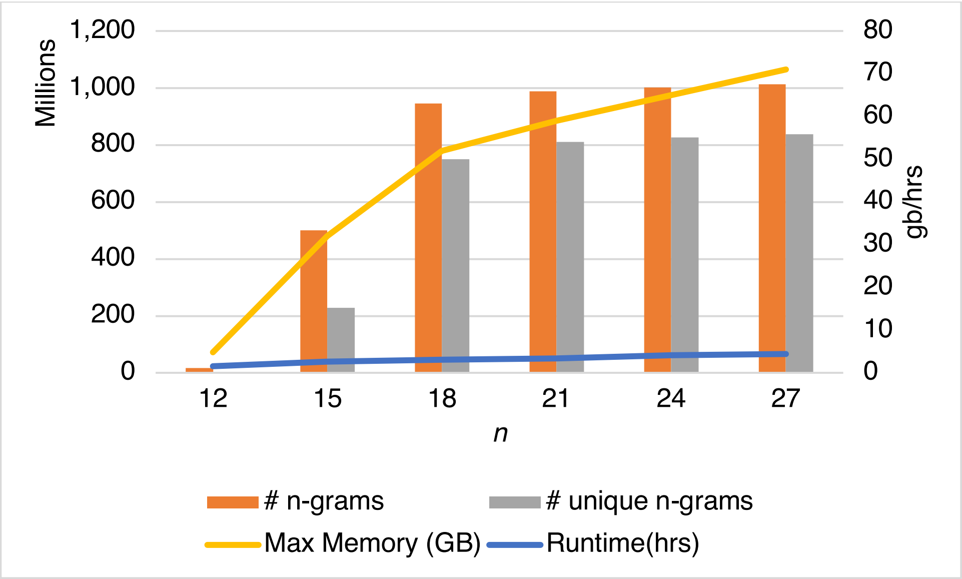

Supplementary Figure 1: Correlation between the n-gram size and number of unique/common n-grams, memory requirement, and runtime using the GI tract reference genomes. The x-axis represents the size of n-gram. The y-axis on the left is the number of n-grams in millions. The y-axis on the right shows the memory in gigabytes (GB) and processing times in hours.

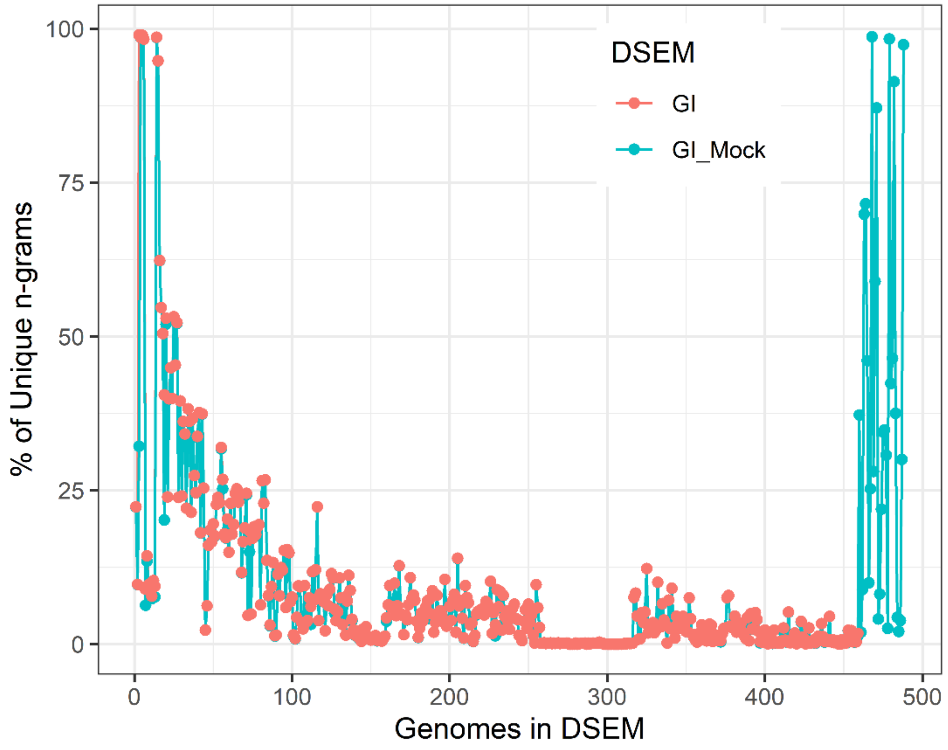

Supplementary Figure 2: Visualization of the percentage of unique *n*-grams in DSEMs with only GI tract genomes (GI DSEM), and with GI tract and mock community genomes (GI Mock DSEM). The green line/dot indicates the unique n-gram percentage in GI DSEM genomes, and the red line/dot indicates the unique n-gram percentage in GI Mock DSEM genomes. In the graph, dots corresponding to smaller unique *n*-gram percentages are overlaid by the larger percentage dots, and the red lines/dot at the right side of the graph correspond to mock community genomes present only in GI Mock DSEM.
